# Working memory in pre-school children with autism spectrum disorder: An eye-tracking study

**DOI:** 10.3389/fpsyg.2022.922291

**Published:** 2022-10-07

**Authors:** Oleg Zacharov, Rene Jürgen Huster, Anett Kaale

**Affiliations:** ^1^Department of Special Needs Education, University of Oslo, Oslo, Norway; ^2^Department of Psychology, University of Oslo, Oslo, Norway; ^3^Norwegian Centre of Expertise for Neurodevelopmental Disorders and Hypersomnias, Oslo University Hospital, Oslo, Norway

**Keywords:** working memory, pre-school children, autism spectrum disorder, typical development, nonverbal mental age, eye-tracking, A-not-B task

## Abstract

Working memory (WM) was examined in pre-school children with Autism spectrum disorder (ASD) and children with typical development using eye-tracking technology. The children were presented with a digital A-not-B task (with a short and a long waiting condition) where they passively viewed animations of a moving train. Moreover, the current study investigated the relationship between non-verbal mental age (NVMA) and the performance on the task. No group differences were found in the average looking durations between the ASD and typically developing (TD) groups on either the short or long waiting conditions. Although the NVMA of the ASD group was lower than that of the TD group there were no correlations between NVMA and task performance in either group. The results suggest that WM in young children with ASD might not be different from that of TD children. However, the results might be due to ceiling effects of the task and thus needs to be further investigated.

## Introduction

Autism spectrum disorder (ASD) can be described by problems in social communication and interaction, and restricted and repetitive behaviors and interests (American Psychiatric Association, 2013). ASD can be reliably diagnosed at the age of 2, however, it is possible to detect it as early as 18 months of age (Hyman et al., 2020). The symptom presentation and severity vary greatly among individuals with ASD. In addition to the “classical” difficulties exhibited by individuals with ASD, emerging evidence demonstrates that executive functioning (EF) impairment may also be prevalent (Garon et al., 2018).

Executive functioning refers to a number of cognitive abilities that are essential for everyday functioning, including working memory (WM), cognitive flexibility, and inhibitory control (Anderson, 2014). These skills are especially crucial during the pre-school years. For example, Pellicano et al. (2017) reported that individual differences in WM and inhibitory control during the pre-school years were uniquely related to variation in school readiness for both typically developing (TD) children and children with ASD.

Working memory refers to the temporary storage and manipulation of information in the brain (Anderson, 2014). A meta-analysis of studies investigating WM revealed that individuals with ASD exhibited significant WM impairment (Cohen’s *d* = −0.61) compared to the control group (Wang et al., 2017). No association between the WM impairment and age was found. Studies of WM in pre-school ASD has generated evidence demonstrating that pre-school autistic children exhibit impairments on various performance-based WM tasks and rating scales. Edmunds et al. (2021) measured WM in children from 2 to 4 year using three tasks; the Hide and Seek Task, the Boxes Task and the Self-Ordered Pointing Task (SOPT). They found that 2–4-year old children with ASD exhibited lower composite scores, combining all three WM tasks, compared to TD children. Similarly, the aforementioned study by Pellicano et al. (2017) demonstrated that 4 1/2–6-year-old children with ASD scored lower than TD children on the Corsi Blocks task measuring WM. In the Corsi Blocks task children are asked to repeat the tapping of blocks in the same order previously displayed by the experimenter.

However, in parallel to studies reporting group differences, there is also evidence showing that WM is intact in pre-school children with ASD. For example, Gardiner et al. (2017) reported no significant differences in WM performance between 3 1/2 and 7-year-old children with ASD and TD children on the Boxes task. In this task, children were asked to find Jack-in-the-box while keeping in mind boxes they already had searched. Also, no WM deficit was found on the Spinning Pots task between 4 and 6-year old TD children and children with ASD (Valeri et al., 2020). In the Spinning Pots task, children are asked to place an object in eight pots which are then covered and rotated. After each rotation the cover is lifted and the child is asked to find the placed object. This procedure is done until 8 objects are found or after 15 trials.

One of the factors that may contribute to the inconsistent findings could be associated with different parameters of the WM tasks. In pre-school ASD, WM has been predominantly investigated with performance-based tasks which vary in administration procedures and levels of difficulty. During administration of most WM tasks, pre-school children are required to understand and comply with verbal instructions, and produce some kind of motor responses, such as pointing. This may, in addition to the WM demands, further challenge children’s mental capacity and thus may negatively influence their performance. Moreover, pre-school children with ASD often have language difficulties which may, despite their potentially intact WM, prevent them from understanding the instructions. Additionally, WM tasks developed for pre-schoolers vary in difficulty levels and may thus elicit differential performance across the pre-school age range. For example, for the Spinning Pots task, the performance was proposed to improve during the 18–42-month period among TD children (Garon et al., 2018). At 42 months and higher, children were expected to exhibit performance at or close to ceiling. This may explain the absence of group difference in the Valeri et al. (2020) study in which the minimum age of participants was 4 years. In contrast, in the Hide and Seek Task children are asked to place paper animal cutouts on the appropriate boxes. Here, the age range at which children’s performance improves has been shown to be broader, namely 18–60 months (Garon et al., 2014). In the Edmunds et al. (2021) study the Hide and Seek Task was administered to participants between 2- and 4-years. Thus, although there are many WM tasks designed for pre-schoolers, some may capture the children’s impairment in a given study while others may not, because they differ in WM demand, or the overall difficulty level relative to the age group in the study. This has important consequences for the interpretation of findings.

Different administration procedures and levels of difficulty of WM tasks would require children to possess sufficient levels of cognitive functioning. The inconsistent findings could be associated with the large heterogeneity in cognitive functioning among children with ASD. To control for this, researchers usually employ standardized tests to match children on language or some general cognitive ability, most commonly non-verbal mental age (NVMA). There is a general agreement that NVMA or language ability could be mediating factors of EF (Stephens et al., 2018). Doing so, however, restricts the representativeness of the group. Besides, while the NVMA is a preferred matching criterion in most studies with autistic pre-schoolers, there is scarcity of research in the ASD field as to whether NVMA is associated with WM performance. One study by Mungkhetklang et al. (2016) demonstrated that WM contributed to non-verbal problem solving for school age TD children and children with Intellectual Disability. Moreover, a recent study by Stephens et al. (2018) measured NVMA and verbal mental age with Mullen Scales of Early Learning (MSEL) in 6-year-old TD children and found that NVMA was a better predictor of EF that was measured by performance-based tasks. The verbal mental age, on the other hand, was a better predictor of parent-reported EF. Having a sample that has a larger spread in NVMA, not only would increase the sample representativeness, but could also shed some light on whether there is a relationship between NVMA and performance on WM tasks in pre-school children. A relationship would suggest that WM impairment could be related more to the general cognitive profiles of children, specifically the NVMA, rather than the diagnosis.

In recent years there has been a growing interest in the use of eye-tracking technology as tool to provide insight into psychological processes. By measuring where a person is looking, one can uncover essential information about how stimuli are being experienced, prioritized, and processed. There are two advantages for using eye-tracking on pre-school children with ASD. First, due to its millisecond-level precision, one can obtain a more precise measure of their attentional patterns. Second, eye-tracking is non-invasive and allows for data to be collected when children passively view the screen. Hence, this makes it an appropriate tool for investigating areas of interest also in minimally verbal and developmentally delayed pre-school children with ASD who often experience difficulties in dealing with complex cognitive demands. Research has shown that development of language in toddlerhood is important for the emergence and development of EF during early childhood (Stephens et al., 2018). In a study by Hill et al. (2015) 5–8-year-old ASD children with and without developmental language disorder (DLD) and a group of children with only DLD were assessed on verbal and non-verbal (visual) WM tasks. The autistic children with concurrent DLD performed worse on the verbal WM tasks than those without DLD. The children with ASD and DLD performed similar to the children with only DLD on most verbal WM tasks. However, there were no group differences in performance on non-verbal WM tasks, which suggests that increased demands associated with verbal WM task could lead to differential performance between ASD children with and without DLD.

The majority of WM tasks require verbal comprehension (i.e., understanding task instructions) for their completion. Developmental and language delays are common in young autistic children (Friedman and Sterling, 2019). Selecting a narrow sample capable of performing on certain tasks, which is usually the case in most studies, ignores those individuals who would more accurately represent the population at this age period. Using a task with removed verbal demands while still capable of assessing WM could be essential in such cases. Moreover, since eye-tracking could be suited for studying children with various cognitive functioning, which assumes varying levels of NVMA, it makes it possible to investigate the relationship between the measured WM performance and NVMA.

Most of the eye-tracking studies that are conducted on children with ASD have focused on social impairment, such as allocation of attention to social stimuli and predictive gaze (Hamner and Vivanti, 2019). There is a scarcity of eye-tracking research investigating EF in individuals with ASD, and to our knowledge only Fanning et al. (2018) has applied this technology to look at WM in pre-school children with ASD. In this study where they used an eye-tracking version of the A-not-B task, children were presented with a character in the middle of the screen for 3 s that then disappeared either into the left or right target location as a sound was played (croaking frog). After a 6 s waiting period another sound (croaking frog) was played for 3 s, implicitly asking children “from which location, A or B, did the sound originate?” It was reasoned that if children memorized which of the two locations the stimulus had disappeared to, on hearing the sound they would fixate at that location. Looking durations to the target and non-target locations were measured during the 3 s after the 6 s waiting period. WM performance was indicated by the duration of the preferential location at the target location. Researchers found no group differences on the eye-tracking version of the A-not-B task between 2- and 5-year-old TD children and children with ASD.

Similarly to the difficulty levels associated with various performance-based WM tasks, caution should also be applied to tasks using eye-tracking. Given that the A-not-B task originally was developed for infants, it is important to adjust the task to the range of the study participants’ developmental level. A more elaborate version of the task might make group differences visible. For example, by introducing shorter and longer waiting periods it may be possible to manipulate the WM load, which will consequently influence the performance.

Overall, given the scarcity of eye-tracking research on WM in pre-school children with ASD and the aforementioned issues that may be associated with task parameters in previous studies, further investigation is necessary. Moreover, since eye-tracking allows for inclusion of developmentally delayed and/or minimally verbal children, it may shed some light on the involvement of NVMA in WM task performance, that would otherwise be difficult to do due to complex cognitive demands associated with manual and verbally loaded tasks.

### Rationale

The aim of the current study is twofold. First the study will investigate and compare the WM performance of pre-school age children with ASD and TD on a novel computerized A-not-B task using the benefits of eye-tracking technology. Since the original A-not-B task is designed for infants and required children to reach for a hidden object, a number of modifications have been made. We kept the task passive without verbal instructions, but incorporated distractors and two WM load conditions (short vs. long waiting period). It is hypothesized that the long waiting period, which poses higher WM load, would affect the performance of children with ASD the most.

Being the most commonly used matching criterion in research on ASD, the NVMA is rarely studied along the EF performance in pre-school children with ASD. While matching provides control for variables that may affect the outcome, little is known whether NVMA could be related to WM performance. Hence, the current study will also investigate the relationship between children’s NVMA and their performance on the A-not-B task. Considering the aforementioned research by Mungkhetklang et al. (2016) and Stephens et al. (2018), it is hypothesized that NVMA of both children with and without ASD would be associated with their performance on the eye-tracking version of the A-not-B task.

## Materials and methods

### Participants

Thirty-seven pre-school children participated in the study. 13 children, aged 31–68 months (*M* = 53.54, SD = 11.22), were diagnosed with ASD, and 24 children, aged 37–59 months (*M* = 49.50, SD = 6), were TD. There were 11 boys (84.6%) and 2 girls (15.4%) in the ASD group, and 11 boys (45.8%) and 13 girls (54.2%) in the TD group (Table 1). The recruitment of children with ASD was done through specialist health services, educational-psychological services, and pre-schools in Oslo and surrounding counties. The recruitment of TD children was done via pre-schools in Oslo and surrounding counties. The children in the ASD group were diagnosed by the specialist health services using the International Classification of Diseases, 10th version (World Health Organization [WHO], 1993) based on a detailed clinical evaluation, including tests, interview with caretakers and observations. As a part of the current study the parents scored their children on the Social Communication Questionnaire (SCQ) (Rutter et al., 2003). All but one child scored above the cut-off for ASD. However, SCQ data from 4 children (30.8%) are missing. Children with severe motor, visual or hearing problems did not participate in the current study. Also, due limitations in eye-tracking technology, children wearing glasses were not included in the study. The Regional Committees for Medical and Health Research Ethics approved the study and written informed consent was given by all parents.

**TABLE 1:** Descriptive statistics for the typically developing (TD) and autism spectrum disorder (ASD) groups.

	ASD (*n* = 13)	TD (*n* = 24)	*t*	*p*	Hedges’ *g*
CA (months)					
*M* (SD)	53.54 (11.22)	49.50 (6)	1.43	0.160	0.49
Range	31–68	37–59			
Social Communication Questionnaire–Parents					
*M* (SD)	16.33 (6.06)				
Range	8–27				
Missing data	4 (30.8%)				
NVMA (Months)					
*M* (SD)	31.58 (10.26)	48.96 (8.57)	−5.37	*p* < 0.001	1.90
Range	14–50	30–67			
Missing data	1 (7.7%)	–			
Receptive Language–Age (months)					

			**Mann–Whitney U**		
			
			***U***	***p***	**η 2**

*M* (SD)	23.67 (11.92)	41.42 (4.8)	27.5	*p* < 0.001	0.44
Range	8–46	30–48			
Missing data	1 (7.7%)	–			
Expressive Language—Age (Months)					
*M* (SD)	25.17 (18.9)	54.75 (13.38)	35.5	*p* < 0.001	0.38
Range	4–67	23–70			
Missing data	1 (7.7%)	-			
Child’s Spoken Language					
Norwegian	7 (53.8%)	13 (54.2%)			
Norwegian + Other	1 (7.7%)	5 (20.8%)			
Missing data	5 (38.5%)	6 (25%)			
Gender					
Male	11 (84.6%)	11 (45.8%)			
Female	2 (15.4%)	13 (54.2%)			
Maternal Education					
Primary School	1 (7.7%)	–			
High School	2 (15.4%)	1 (4.2%)			
University	8 (61.5%)	17 (70.8%)			
Missing data	2 (15.4%)	–			

### Procedure

The present research was a part of a longitudinal project which aim was to study early development and learning in TD children and children with ASD. All children were tested during 1 day with multiple measures of language and cognitive ability. WM was measured with the eye-tracking version of the A-not-B task. The task was built in Experiment Builder (SR Research) and presented on the Lenovo ThinkPad W550s laptop. All children were seated approximately 70 cm from the laptop screen. Eye-tracking data was recorded with EyeLink 1000 Plus. No chin rest was used during the recording. Instead, a target sticker was placed on the children’s forehead or cheek allowing the participants to move during the recording while the eye-tracking camera followed their eyes. The eye-tracking recording was performed on the left eye (monocular) for all participants. The sampling rate was set to 500 frames per second. A 16 mm camera lens was used. Children performed the task without familiarization phase. The experiment could be paused at any moment during the testing. During the pause, a flickering image could be played on the presentation laptop in order to attract the child’s attention back to the screen. After the pause, the previous trial was restarted. The sound stimuli were played through external speakers. All the testing, including the eye-tracking, was carried out by the first author and a research assistant in a quiet room in the children’s pre-school or in the laboratory at the University of Oslo. Test duration for each child ranged from 2 to 4 h including breaks. Social (e.g., praise, play brakes) and edible motivators (e.g., raisins, apple bits) were provided when necessary to increase children’s compliance. As the eye-tracking WM task require the child to be attentive to the screen it was usually administered on the first hour of testing. All parents were asked to fill out questionnaires in order to obtain demographic information.

### Measures

#### Cognitive and language level

Children’s NVMA and expressive and receptive language were measured with the (MSEL; Mullen, 1995). Infants and children up to 68 months of age are eligible for MSEL. MSEL consists of five subscales, namely Gross Motor, Fine Motor, Expressive Language, Receptive Language, and Visual Reception. Composite of the Visual Reception and Fine Motor subscales were used to calculate the NVMA, while the Receptive and Expressive subscales were used to calculate language level.

### Working memory measure

#### A-not-B task

The current task is a modification of the manual version of the A-not-B task. Although the task was initially proposed to measure WM in infants and toddlers (Diamond et al., 1997), original and modified manual A-not-B tasks have also been used with pre-school autistic children aged between 40 and 80 months of age (McEvoy et al., 1993; Griffith et al., 1999). The modifications were made to the original task in order to increase WM load. In the manual version of this task, the child is presented with two containers. During “A” trials, a toy is hidden in the container “A” and the researcher leaves the child to find the toy, usually by asking the question “where is the toy?” After a number of consecutive “A” trials, the toy is then hidden in the container “B.” After hiding the toy during “B” trials, the experimenter claps his/her hands in order to disengage the child’s attention from the hiding area. Afterward, the child is allowed to search for the toy. Infants and toddlers usually make a perseverative error during this trial by continuing to search for the toy in the container “A” (Diamond et al., 1997).

**FIGURE 1:**
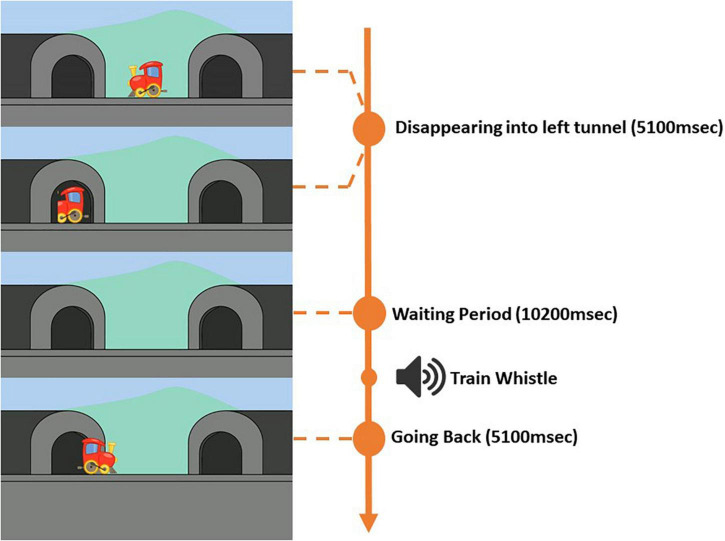
Progression of the A Trial.

In the current task, children viewed a series of movie clips (width = 1,920, height = 1,080, frame rate = 25) of a train moving from the center into a tunnel either to the left or the right. The train moving into the left tunnel constituted “A” trials, and the train moving into the right tunnel constituted “B” trials. In total, there were 10 trials, six “A” trials and four “B” trials. The trials were presented in the following order: “A,” “A,” “A,” “B,” “B,” “A,” “A,” “A,” “B,” “B.” Each trial (“A” and “B”) was separated into three parts: (1) a train going and disappearing into the tunnel, (2) a waiting period with (“B” trials) or without (“A” trials) distractor, and (3) the train returning back to center. A melody was played throughout the task. It was possible to pause the task at any moment and repeat an interrupted trial. As illustrated in Figure 1, at the beginning of each “A” trial, the train that was positioned at the center of the screen (*x* = 960, *y* = 540) would start moving horizontally to the left side of the screen and disappearing into the tunnel (*x* = 340, *y* = 540). The time it took for the train to disappear completely into the tunnel was 5,100 ms. A chugging train sound (duration = 5,100 ms) was played as the train moved toward and disappeared into the tunnel. After the train had disappeared, a waiting period was initiated. For “A” trails, the waiting period was 10,200 ms. 5,100 ms into the waiting period, the train whistle sound was played for 1,870 ms followed by a chugging train sound that was played for the rest of the waiting period and until the end of the “A” trial. The train whistle sound was implemented to signal the children that the train was about to come back from the tunnel. After the waiting period the train reappeared and moved back from the same tunnel to the center of the screen. Each “A” trial lasted in total for 20,400 ms. For “A” trials, the left tunnel was the correct target location. A 5-point calibration and validation procedure preceded the task.

**FIGURE 2:**
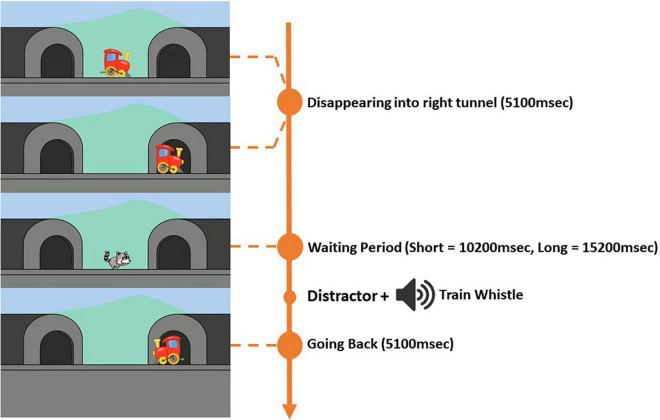
Progression of the B Trials with short (10,200 ms) and long (15,200 ms) waiting periods.

As for the “B” trials (Figure 2), there were two trial types, one with a shorter waiting period (10,200 ms) and one with a longer waiting period (15,200 ms). In total, there were two “B” trials with a shorter period and two “B” trials with a longer waiting period. The same trial types were never presented in succession. At the beginning of each “B” trial, a centrally positioned train (*x* = 960, *y* = 540), would start moving horizontally to the right side of the screen and disappearing into the tunnel (*x* = 1,580, *y* = 540) within 5,100 ms. As for the “A” trials, an accompanying chugging train sound would play for 5,100 ms until the train had completely disappeared. Depending on the trial type, a short or long waiting period was then initiated. Both “B” trials types had distractors presented during the waiting period. The distractors were in the form of moving animations that were located in the center of the screen. Each “B” trial had a different distractor. The distractors for the “B” trials were always presented at 1,000 ms into the waiting period, and were present for 4,100 ms. The distractors were implemented to divert attention and prevent children from fixating at the tunnel into which the train had disappeared for the rest of the waiting period. A train whistle sound was then played for 1,870 ms at 5,100 ms for “B” trials with a shorter waiting period and at 10,100 ms into the waiting period for “B” trials with a longer waiting period. A chugging train sound was then played for the rest of the waiting period and until the end of the “B” trial. After the waiting period the train would reappear from the same tunnel and move back to center of the screen within 5,100 ms. The total duration of the “B” trial with a shorter waiting period was 20,400 ms and the total duration of the “B” trial with a longer waiting period was 25,400 ms. For “B” trials, the right tunnel was the correct target location.

The eye-tracking version of the A-not-B task relies primarily on the voluntary sustained anticipatory looking until the appearance of the stimulus at the location where the participant expects it to appear. The addition of distractors was necessary to increase the difficulty of the A-not-B task (Watanabe et al., 2012). Moreover, “B” trials with a longer waiting period were incorporated to increase the demand for WM thus increasing the task’s difficulty. These features were implemented in an attempt to make the task more appropriate for pre-school age children.

### Data and statistical analysis

**FIGURE 3:**
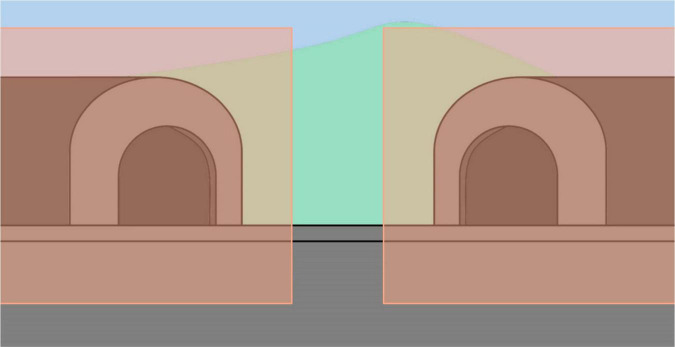
Two identical Interest Areas for the left and right tunnels.

Descriptive data [e.g., chronological age (CA), NVMA, language level] were analyzed for both groups and reported as means, standard deviations, ranges or frequency and percentages. The eye-tracking data was preprocessed in Data Viewer (SR Research). Two areas of interest were created, equally encompassing both the left and right tunnels (Figure 3). The data from “A” trials was used primarily from TD children to test whether children would spend more time looking at the target location which would insinuate that the task was understood. Similarly to Fanning et al. (2018), it was speculated that if the location of the disappeared train was remembered, children would look longer at the correct tunnel upon hearing the train whistle sound stimulus during the waiting period. Fanning et al. (2018) tested this prediction in a pilot study where a group of TD children exhibited greater preferential fixation to the target versus non-target area. This prediction was also tested in the current study using data from the TD pre-school children. A *t*-test revealed that the TD children (*n* = 23) spent significantly more time looking at the left tunnel (*M* = 2,122 ms, SD = 666.82) than the right tunnel (*M* = 1,150 ms, SD = 470.95) (*t*(24) = 5.195, *p* < 0.001, 95% CI[584.79–1,358.67]).

For both “B” trials types (short and long waiting period), the dependent variables were the average looking durations at the left and the right tunnels during a 5-s period from the start of the train whistle sound to the reappearance of the train from the tunnel into which it has disappeared. The averages were made separately for the “B” trials with shorter and longer waiting periods. This way, it was possible to measure children’s “correct” and “incorrect” anticipatory looking after the change of the hiding location. Similar to previous research, only fixations of 100 ms and longer were included in the analyses (Fanning et al., 2018). The average looking durations are reported in milliseconds.

First, for both “B” trial types, paired samples *t*-tests were used to investigate within group differences in looking durations between the left (incorrect) and right (correct) tunnels. Second, independent *t*-tests were conducted to compare looking durations at the right tunnel in the two types of “B” trials between the children with ASD and the TD children. Next, for each group, paired samples *t*-tests were conducted to investigate whether there was a statistical difference in looking durations at the right tunnel between “B” trials with shorter and longer waiting periods. To account for multiple tests, a Bonferroni correction was applied. An adjusted alpha level of 0.01 for statistical significance was used. Last, to investigate the relationship between NVMA and the average looking durations in the two groups during the two “B” trial types, a series of Spearman’s correlation analyses were performed. One child from the ASD group was not included in the correlation analysis due to missing NVMA data. The data was analyzed in Statistical Package for the Social Sciences (SPSS) 27.

## Results

### Group difference on “B” trials with shorter waiting period

**FIGURE 4:**
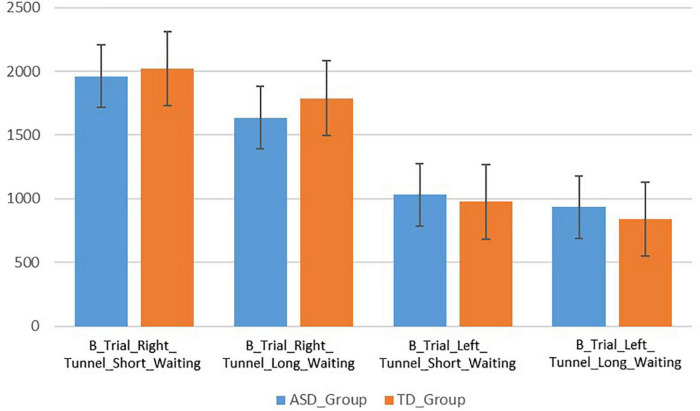
Bar graph showing the looking durations at the right and left tunnels in milliseconds on B trials with short and long waiting periods for both groups.

The children with ASD spent more time looking at the right tunnel (*M* = 1,961, SD = 1,096) than at the left tunnel (*M* = 1,032, SD = 469) during the B trials with shorter waiting period (Figure 4). This difference was statistically significant (*t*(12) = −2.92, *p* = 0.013, 95% CI[236–1,620]). Similarly, the TD children spent more time looking at the right tunnel (*M* = 2,020, SD = 868) than at the left tunnel (*M* = 975, SD = 620) (Figure 4). This difference was also significant (*t*(23) = −3.98, *p* = 0.001, 95% CI[502–1,588]). There were no significant differences in looking durations at the right tunnel between the ASD and TD groups (*t*(35) = −0.181, *p* = 0.857, 95% CI[−606–725], Hedges’ *g* = 0.061).

### Group difference on “B” trials with longer waiting period

The ASD group spent slightly more time looking at the right tunnel (*M* = 1,635, SD = 1,105) than at the left tunnel (*M* = 933, SD = 1,003), but the difference was not statistically significant (*t*(12) = −1.95, *p* = 0.074, 95% CI[−78.65–1,483]). The TD group spent more time looking at the right tunnel (*M* = 1,788, SD = 992) than at the left tunnel (*M* = 839, SD = 487). This difference was significant (*t*(23) = −3.72, *p* = 0.001, 95% CI[421–1,475]). There were no significant differences in looking durations at the right tunnel between the ASD and TD groups (*t*(35) = −0.429, *p* = 0.671, 95% CI[−569–874], Hedges’ *g* = 0.148).

### Within group difference on “B” trials with shorter versus longer waiting period

For the ASD group, there was no statistical significance difference in looking durations at the right tunnel between “B” trials with shorter and longer waiting periods (*t*(12) = 1.296, *p* = 0.219, 95% CI[−221.99–873.30]). For the TD group, there was no statistical significance difference in looking durations at the right tunnel between “B” trials with shorter and longer waiting periods (*t*(23) = 1.466, *p* = 0.156, 95% CI[−95.68–560.85]).

### Relationship between non-verbal mental age and “B” trials with shorter and longer waiting period

No correlations were found between NVMA and looking durations at the right tunnel for either the children with ASD or TD on the trials with shorter and longer waiting periods (Table 2).

**TABLE 2:** Correlations between non-verbal mental age (NVMA) and looking durations for “B” trial with short and long waiting period for both groups.

	ASD (*n* = 12^1^)	TD (*n* = 24)
**Variable**	**NVMA**	**NVMA**
≪B≫ Short Waiting Period–Target Looking	−0.371	−0.017
≪B≫ Long Waiting Period–Target Looking	−0.046	−0.052

^1^Data from one of the 13 children missing.

## Discussion

The current study compared WM performance on an eye-tracking version of the A-not-B task between pre-school children with ASD and TD children. The group comparisons were performed on “B” trials with short and long waiting periods. Average looking durations were used as dependent variable. On average, both the ASD and TD groups spent significantly more time looking at the target location (right vs. left tunnel) during “B” trials with short waiting periods. Only the TD groups spent significantly more time looking at the target location during long waiting period. There were no significant differences in average looking durations to the right tunnel between the two groups during the tasks with either the short or the long waiting period. Moreover, neither group exhibited significant difference between looking durations at the target location during shorter vs. longer waiting period. This suggest that the introduction of a longer waiting period had no effect on looking durations at the target location. The results are contrary to the initial expectation that children with ASD would exhibit shorter looking durations at the target location than the TD group on both “B” trial types, and that the group differences would be larger in the condition with longer waiting period due to its higher WM demand. Complementary to the study by Fanning et al. (2018) that also found no significant differences between 2- and 5-year-old TD children and children with ASD on the eye-tracking A-not-B task, neither the addition of distractors nor a condition with longer waiting period in the current study lead to the identification of differences between the groups.

The current study also investigated the relationship between NVMA and average looking durations during the “B” trials with short and long waiting periods between the ASD and TD groups. As expected, the children in the ASD group had substantially lower NVMA as compared to the TD group. Still, correlations between NVMA and performance on “B” trials were not identified in either group.

The lack of group differences and correlation to NVMA could be related to children’s developmental level, which possibly have exceeded the task’s age range for which it is suited. The A-not-B task was previously used with children younger (15–30 months) than the current sample (Diamond et al., 1997). Studies by Griffith et al. (1999) and McEvoy et al. (1993) had samples with ages ranging from 40 to 80 months old performing the modified manual A-no-B tasks. However, no group differences were found in those studies. In the current sample, the mean NVMA of the ASD group was 31.5 months and the mean NVMA of the TD group was 48.9. Although an attempt was made in increasing tasks difficulty by implementing distractors and different waiting periods, the NVMA of children in the current study might be above the task’s targeted age range.

Although eye-tracking provides the benefit of acquiring data from children who are minimally verbal and/or developmental delayed, it is important to ensure that the task targets the participants’ developmental level. The aforementioned study by Valeri et al. (2020) in which 4–6-year old children with ASD displayed no impairment on Spinning Pots task which was proposed to be suitable for children between 18 and 42 months could act as an example of the importance of task selection. Additionally, in a study by Zacharov et al. (2021) pre-school children with ASD were administered two cognitive flexibility tasks designed for pre-schoolers. These tasks had different difficulty levels. On the more difficult task the children exhibited impairment, while on the other their performance was intact.

Small sample size is one of the main weaknesses of the current study. Thus, the interpretation of the results should be done with caution. Additionally, the gender ratio of the ASD sample is skewed, hence no potential gender differences could be investigated. However, the current study demonstrated that it is possible to measure WM with a cartoon-like task consisting of different WM loads using eye-tracking technology. Moreover, the current study further illuminated the important issue of task appropriateness when studying EF in pre-school children with ASD. The current task may be suitable for even younger children with ASD and TD. Hence, in future studies employing a similar eye-tracking version of the A-not B task, it is recommended to recruit younger participants than in the current sample.

## Data availability statement

The datasets presented in this article are not readily available due to restrictions related to ethical regulations. Requests to access the datasets should be directed to AK, anett.kaale@isp.uio.no.

## Ethics statement

The studies involving human participants were reviewed and approved by the Regional Committees for Medical and Health Research Ethics (REC), South East Norway. Written informed consent to participate in this study was provided by the participants’ legal guardian/next of kin.

## Author contributions

OZ conducted the data collection. All authors contributed in writing the manuscript, took part in the design and analyses of this study, and approved the submitted version.
